# TSLP: contrasting roles in cancer

**DOI:** 10.3389/fimmu.2025.1627235

**Published:** 2025-08-12

**Authors:** Remo Poto, Gianni Marone, Steven F. Ziegler, Gilda Varricchi

**Affiliations:** ^1^ Department of Translational Medical Sciences, University of Naples Federico II, Naples, Italy; ^2^ Istituti Clinici Scientifici Maugeri-Scientific Institute for Research Hospitalization and Healthcare (IRCCS) Scientific Institute of Telese Terme, Benevento, Italy; ^3^ World Allergy Organization (WAO), Center of Excellence (CoE), Naples, Italy; ^4^ Center for Basic and Clinical Immunology Research (CISI), University of Naples Federico II, Naples, Italy; ^5^ Benaroya Research Institute, Seattle, WA, United States; ^6^ Division of Allergy and Infectious Diseases, University of Washington, Seattle, WA, United States; ^7^ Department of Immunology, University of Washington, Seattle, WA, United States

**Keywords:** alarmin, cancer immunity, cytokine, TSLP, TSLP isoforms, tumor microenvironment

## Abstract

Thymic stromal lymphopoietin (TSLP) is an alarmin cytokine possessing a plethora of pleiotropic properties. Human and mouse TSLP exerts their activity *via* a heterodimeric complex composed of TSLP receptor (TSLPR) chain and IL-7Rα. TSLP is predominantly expressed by epithelial cells and keratinocytes but can also be produced by several immune cells and some cancers. TSLP activates a plethora of immune cells implicated in inflammation, angiogenesis and tumorigenesis. In addition to its role in barrier immunity, recent studies have a role for TSLP in cancer development. This includes both human hematologic cancers and several solid tumors (largely carcinomas). The role of TSLP in human and experimental cancers has been the focus of several studies, with somewhat contradictory findings. In this Review, we will highlight recent advances in TSLP immunobiology in the context of human and experimental cancers. We will also discuss recent findings demonstrating that an anti-TSLP monoclonal antibody (mAb) can exert a protective effect in a mouse model of colorectal cancer. The recent approval of an anti-TSLP mAb for asthma treatment also emphasizes the urgent need for additional research on the role of TSLP, a Janus cytokine, in tumorigenesis.

## Introduction

**Figure 1:**
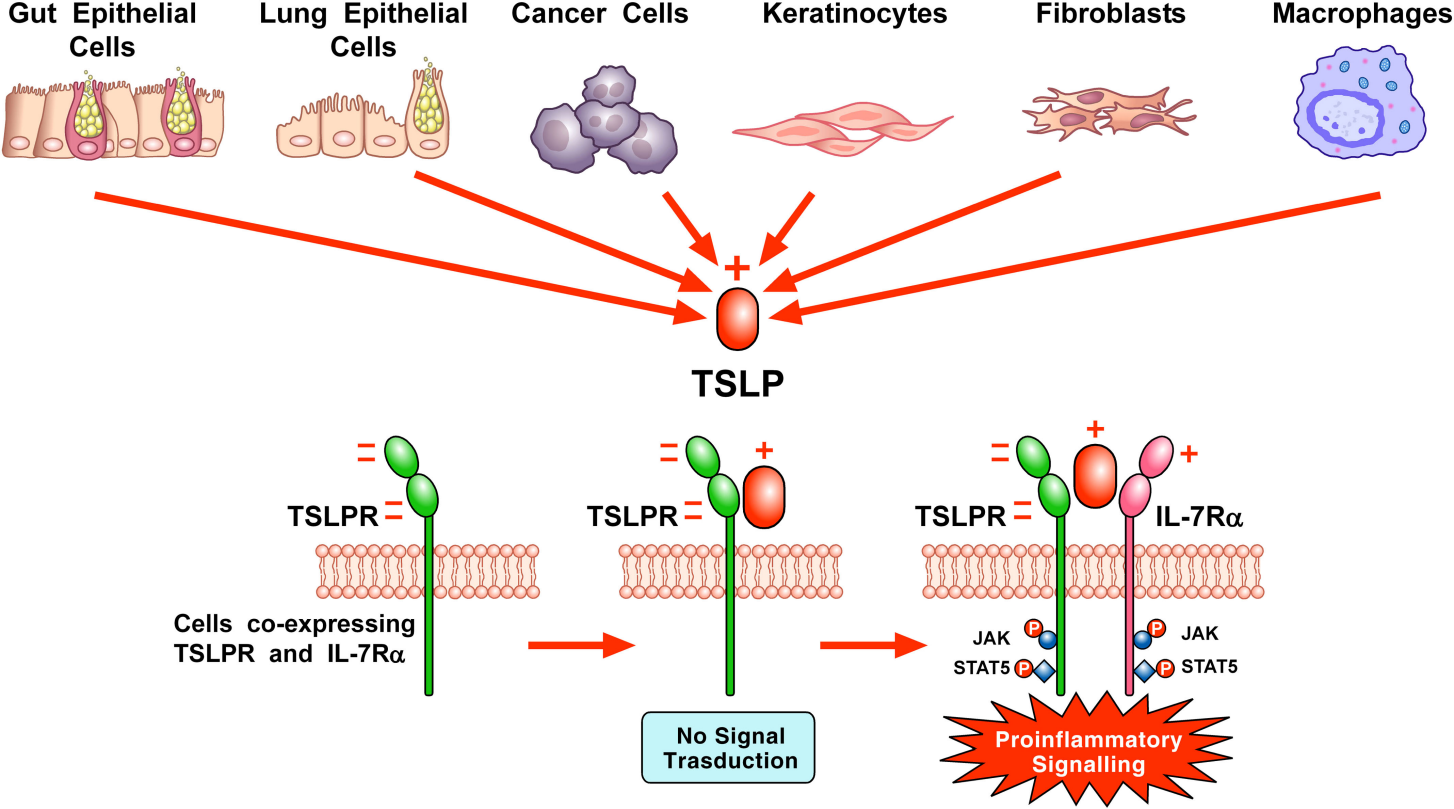
Schematic representation of TSLP-mediated receptor activation and signaling on the surface of cellular targets. A variety of triggers (e.g., cytokines, cigarette smoke extracts, viral, bacterial and fungal products, allergens, and tryptase) can stimulate the production and release of TSLP by lung and gut epithelial cells, cancer cells, keratinocytes, and/or macrophages. TSLP, positively charged, binds to the highly negatively charged TSLP receptor (TSLPR). Then, IL-7Rα, positively charged, can be recruited to the TSLPR: TSLP binary complex to form the ternary TSLPR-TSLP-IL-7Rα complex (11). This receptor complex phosphorylates Janus kinases (JAKs) and signal transducers and activators of transcription 5 (STAT5) to initiate proinflammatory signaling in target cells (Adapted from Varricchi et al., Front. Immunol 2018).

Thymic stromal lymphopoietin (TSLP) is a member of the 4-helix bundle cytokine family, and a distant paralog of IL-7 (1). As the name suggests, TSLP was first identified in the supernatant of a mouse thymic stromal cell line for its activity in supporting immature B cell proliferation and development (2–4). A human TSLP homolog was subsequently identified in humans using *in silico* methods (5, 6). Several groups isolated a TSLP-binding protein in both humans and mice [referred to as TSLP receptor (TSLPR) in mice and cytokine receptor-like factor 2 (CRLF2) in humans] (7–10). Sequence analysis found that TSLPR was most closely related to the common gamma chain (γc) (7). It is now known that the functional, high affinity, TSLPR complex is a heterodimer of TSLPR and interleukin 7 receptor alpha (IL-7Rα; **Figure 1**) (7, 8). Cross-species homology for both the cytokine and its receptor is relatively low (~40% for each), although functionally they appear to be quite similar. Thus, the role of this cytokine axis is conserved between human and mouse despite of a loss of sequence identity.

A primary cellular target for TSLP are dendritic cells (DCs), which upregulate OX40L, CD80, and CD86 in response to TSLP, and TSLP-treated DCs can drive IL-4, IL-5, and IL-13 production from naïve CD4^+^ T cells upon co-culture (12–15). In addition to its effects on Th2 cell polarization through antigen-presenting cells, TSLP can also act directly on CD4^+^ and CD8^+^ T cells, and Treg cells (16–18). TSLP can also promote Th2 cytokine responses through its actions on mast cells, innate lymphoid cells (ILCs), epithelial cells, macrophages, and basophils (19–23). Finally, TSLP was found to play an important role in mouse basophil biology, where *in vitro*, TSLP could induce basophil maturation from bone marrow precursors in an IL-3 independent manner. Furthermore, TSLP-elicited basophils *in vivo* were phenotypically distinct from IL-3-elicited basophils (24).

TSLP is expressed at basal levels at mucosal surfaces (e.g., gut and lung), as well as in the skin (5, 25–27). Its expression can be further enhanced through exposure to viral, bacterial, or parasitic pathogens as well as Toll-like receptor (TLR) agonists (22, 28, 29). A link between TSLP expression and atopic disease was first established by Soumelis et al. who showed elevated expression in the lesional skin of individuals with atopic dermatitis (AD) (30). Following that finding, TSLP expression was found in the airways of patients with asthma and in the nasal lavages of individuals with allergic rhinitis (31–33). TSLP levels in asthmatic airways correlated with Th2-attracting chemokine expression and disease severity (33). In eosinophilic esophagitis (EoE), a gain-of-function polymorphism in TSLP is associated with disease in pediatric subjects (34, 35), and TSLP expression was higher in esophageal biopsy samples from children with active EoE compared to subjects with inactive EoE (36).

Historically, physicians have noted that Type(T)-2 inflammatory disorders often develop in an individual patient in a typical sequential order, with AD occurring first, followed by food allergy and then upper and lower airway disease (37). This sequence, often referred to as the “atopic march” (38), highlights the potential role of TSLP and the other epithelial cytokines as initiators and propagators of allergic disease. Studies over the past 20 years have shown TSLP to be an important driver of the atopic march in both humans and rodents. Previous clinical and experimental studies concluded that the role of TSLP-TSLPR axis in cancer was controversial (39–41). Since then, several experimental and clinical studies have shed light on the different mechanisms of the protumorigenic role of TSLP and its isoforms in cancer. In this Review, we will summarize the work on TSLP immunobiology, emerging data regarding TSLP isoforms and a new-found role for TSLP in a wide variety of cancers.

## TSLP in type-2 inflammation

Epithelial-derived cytokines, including TSLP, IL-33, IL-25, and TL1A, play critical roles in the development of allergic responses at barrier surfaces (42). These alarmins have been implicated in the pathogenesis of T2 inflammatory diseases, including AD (43), food hypersensitivity reactions (44), asthma (45, 46), CRSwNPs (47) and chronic obstructive pulmonary disease (COPD) (48, 49). The release of these alarmins is stimulated by epithelial exposure to allergens (particularly those rich in proteases), microbes (viruses, bacteria, parasites), and inorganic chemicals. Although the inducing stimuli, cellular sources, target populations and functions of alarmins share similarities, several differences characterize the three epithelial-derived cytokines (23, 42). Actually, there is some evidence that TSLP and IL-33 can synergistically enhance certain aspects of innate T2 airway inflammation (50).

TSLP has diverse effects in Type 2 (T2) inflammation. The most proximal effect of TSLP in this regard, shared with IL-33, is the upregulation of DC expression of OX40L, CD80, and CD86, which are required for T helper T2 cell (T_H_2) polarization (12). While expression of the IL-33 receptor ST2 on T_H_2 cells requires prior cell activation, TSLPR expression does not require T_H_2 cell activation and can be identified on naïve CD4^+^ T cells (51, 52), suggesting a possibly earlier role for TSLP. There are a number of other significant effects of TSLP on a broad range of cell types, including increased proliferation of T cells (18) and T_H_2 cells (53) and release of T_H_2 cytokines and chemokines from mast cells (54), ILCs (21), and macrophages (55) (48, 56). While the role of TSLP in human basophil activation is controversial (23, 57), mouse basophils appear to play an important role in the induction of TSLP-mediated T_H_2 inflammation (24, 58). Using a mouse model that employed the vitamin D analog MC903 to induce TSLP release from keratinocytes, investigators demonstrated that TSLP-activated DCs prime CD4^+^ T cells *via* OX40L signaling to produce IL-3, leading to recruitment of basophils. As these events precede the induction of IL-4 production by T cells, mouse basophils may provide an initial source of IL-4 early in the course of T_H_2 immune responses, suggesting that this sequential cascade of DCs, T cells, and basophils is critical to T cell expansion and T_H_2 priming.

The clear role of TSLP in atopic diseases led to the development of a neutralizing anti-TSLP human monoclonal antibody, referred to as tezepelumab. Tezepelumab has been used in clinical trials to treat a variety of T2 conditions, including AD (59), EoE (NCT05583227), asthma (60) and chronic rhinosinusitis with nasal polyps (CRSwNPs) (61). In a small study of patients with moderate-to-severe AD, treatment with tezepelumab resulted in a numerical, but not statistically significant improvement in eczema severity scores, likely due to the use of background medication during the trial (59). Tezepelumab has been extensively tested in patients with severe asthma. A large Phase III trial using tezepelumab in severe asthmatics to decrease exacerbations showed a clear benefit in glucocorticoid-resistant asthma compared to the placebo group (60). Importantly, the frequencies and types of adverse events did not differ between the two groups. Based on these results, tezepelumab has been approved by the American FDA in 2021 and the European EMA in 2023 for treatment of severe asthma. Recently, tezepelumab significantly reduced nasal polyp size, nasal symptoms and the need for nasal polyp surgery or systemic glucocorticoids in severe CRSwNPs compared to placebo (61).

## Structural basis of TSLP-mediated receptor activation and signaling

X-ray crystallographic analysis of human TSLP showed that this cytokine has a four-helix bundle structure with four alpha helices (αA, αB, αC, and αD) arranged in an alternating ‘up-up-down-down’ configuration (11, 62). The TSLP four-helix bundle is threaded by three loops (a *BC-, AB*-, and *CD*- loop). Human TSLP contains six cysteine residues forming three disulfide bonds (11, 63).

TSLP engages a heterodimeric complex comprising the TSLPR, a type I cytokine receptor, and IL7Rα, a receptor also engaged by IL-7, on several target cells (7, 8). TSLPR, highly negative, binds TSLP containing several positively charged amino acids with high affinity (K_d_ = 32 nM). Although IL-7Rα does not interact with TSLPR alone, IL-7Rα associates with high affinity (K_d_ = 29 nM) to the TSLP: TSLPR binary complex (11, 62). TSLP binding induces the dimerization of these receptor chains, triggering Janus kinases (JAKs) and signal transducers and activators of transcription 5 (STAT5) signaling, leading to the transcription of genes in several targets cells (5, 6, 64, 65) (**Figure 1**).

The interaction of TSLP with TSLPR (site I) is mediated by electrostatic attraction, with a positively charged region on TSLP interfacing with a negatively charged area on TSLPR. This interaction establishes a binary complex with a negative charge, priming it for the addition of IL-7Rα, which has a positive electrostatic potential. Critical contact points for the amino acids involved in TSLP: TSLPR interactions are located in the C-terminal region of αD helix and *AB*-loop region undergoing conformational changes. The *AB-* loop offers a link to the αA helix, playing a crucial role in the engagement with IL-7Rα at site II. This interaction is essential for conferring an entropic benefit that facilitates the assembly of a stable T-shaped ternary complex. In addition to the αA helix’s role, the hydrophobic surface of IL-7Rα engages with various outward-facing residues on TSLP’s αC helix, further stabilizing the interaction (11).

## TSLP isoforms

**Figure 2:**
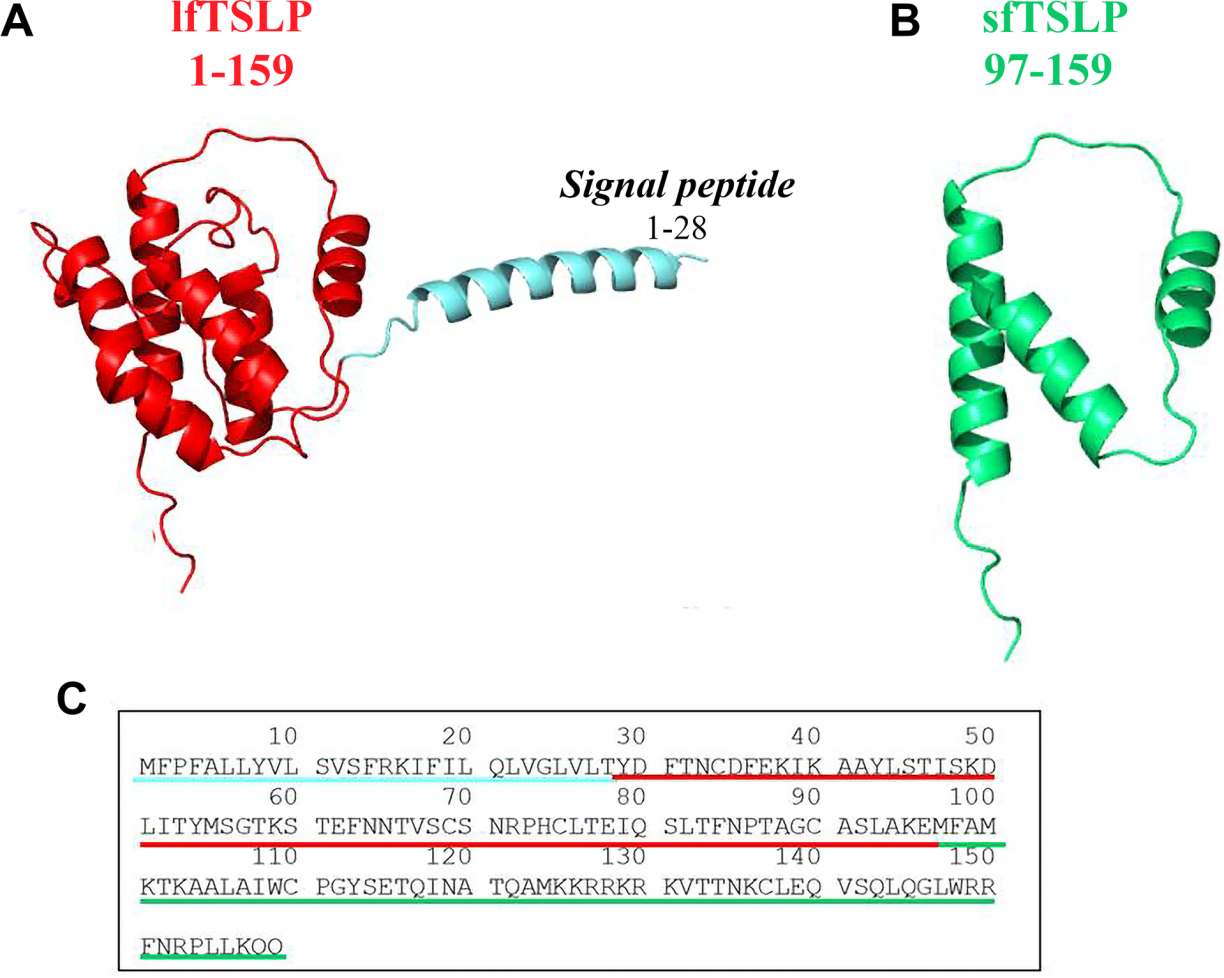
Three-dimensional (3D) structure of human long form TSLP (lfTSLP) and short form TSLP (sfTSLP). **(A)** TSLP is a small protein of 159 amino acids, which has a signal peptide of 28 amino acids at the N-portion of the protein (6). **(B)** The sequence of the 63 amino acids of sfTSLP is homologous to the C-terminal portion of the lfTSLP. **(C)** Amino acid sequence of lfTSLP (underlined in red), the signal peptide (underlined in light blue), and sfTSLP (underlined in green).

Harada et al. first discovered two TSLP isoforms in human bronchial epithelial cells (66, 67). The long form TSLP (lfTSLP), which is the homolog of mouse TSLP, is a small protein of 159 amino acids, which has a signal peptide encoded in the first 28 amino acids at the N-terminal portion of the protein (1, 6) (**Figure 2**). The amino acids sequence spanning 63 residues in the short form TSLP (sfTSLP) shares homology with the C-terminal segment of the long form. The mRNA encoding sfTSLP was shown to be initiated from an internal promoter in intron 2 of the *TSLP* gene (66). The relevance of sfTSLP is unclear for a variety of reasons (49). First, sfTSLP mRNA appears to be human specific and there are no reports of a similar variant in other species (40). Second, while there is evidence that the sfTSLP mRNA is constitutively expressed in a variety of tissues, including bronchial and colonic epithelial cells, keratinocytes, and lung fibroblasts (66, 68–72), there is no evidence for expression of a sfTSLP protein (49). This is further complicated by the lack of anti-sfTSLP antibody reagents. Thus, the biological role, if any, of sfTSLP remains largely unknown. Previous research has largely overlooked the application of analytical methods to investigate the differential expression patterns and roles of the two distinct isoforms of TSLP in different cancers.

There is an additional level of complexity in studying the pathophysiological role of TSLP due to its post-translational cleavage. The protease furin can cleave TSLP, generating fragments of 10 and 4 kDa with different activity on human peripheral blood mononuclear cells compared with the mature cytokine (73). Carboxypeptidase N can also cleave TSLP to form two peptides, which strongly activate human DCs (63). Mast cell-derived tryptase and chymase rapidly cleave TSLP to generate several peptides without apparent biological activity on human lung macrophages (48, 74). These findings emphasize the need for additional studies on the role of post-transcriptionally cleaved products of TSLP in tumor biology.

## Immune cellular targets of TSLP

TSLP can modulate the activation of various immune cell populations, including DCs (11, 63, 75, 76), CD4^+^ T cells and Th2 cells (18, 51). In particular, TSLP signaling in CD4^+^ T cells programs a pathogenic Th2 cell state (77). TSLP limits primary and recall responses of CD8^+^ T cell (78), which play a critical role in cancer immunity (79). TSLP is a critical mediator acting on ILC2s (63, 80, 81), and drives the development of Th2 cells (51). TSLP provides critical signals for human (82) and mouse B cell proliferation (83) and also expands bone marrow B cell precursors to support lung metastasis in a breast cancer model (84). TSLP-activated DCs promotes Tfh differentiation from naïve CD4^+^ T cells (75). Tfh cells are important constituents of tertiary lymphoid structure in human breast cancer (85). Moreover, TSLP influences regulatory T cells (Tregs) (86–88).

Initial studies demonstrated co-expression of TSLP receptor (TSLPR) and IL-7 receptor α chain (IL-7Rα) mRNA in human monocytes, with TSLP stimulation inducing CCL17 production (5). Borriello et al. (89) demonstrated that freshly isolated monocytes do not express detectable levels of TSLPR or IL-7Rα, as assessed by flow cytometry, nor do they exhibit STAT5 phosphorylation in response to TSLP. Exposure to lipopolysaccharide (LPS) induced expression of the TSLPR complex in a subset of monocytes. These results highlighted an unrecognized phenotypic and functional heterogeneity within the human monocyte compartment based on TSLPR expression.

*In vivo* administration of TSLP modulates the differentiation of alternatively activated macrophages (55). Interestingly, TSLP potentiated CCL17 production induced by IL-4 from murine macrophages. We presented novel evidence demonstrating the constitutive intracellular presence of TSLP within the cytoplasm of human lung macrophages (HLMs) (48). Upon stimulation with both type 2 (T2)-high and T2-low inflammatory stimuli, HLMs secreted TSLP (56, 74). Moreover, the long isoform of TSLP (lfTSLP) stimulated the release of vascular endothelial growth factor A (VEGF-A) from HLMs (48). In contrast, the short isoform of TSLP (sfTSLP) neither induced VEGF-A production nor inhibited the stimulatory effect of lfTSLP. These findings reveal a previously unrecognized feedback loop between HLMs and TSLP that may contribute to the regulation of inflammatory and tumor angiogenesis (48, 90).

Both TSLPR and IL-7Rα are expressed at the mRNA and protein levels in CD34^+^ progenitor-derived mast cells as well as in mast cells isolated from human lung tissue (91). TSLP, alone or in combination with proinflammatory cytokines such as IL-1β or TNF-α, did not induce mast cell degranulation or the release of lipid mediators (91, 92). Nonetheless, when co-stimulated with IL-1β or TNF-α, TSLP promoted the secretion of multiple cytokines and chemokines (91, 93, 94). Additionally, TSLP has been shown to enhance prostaglandin D_2_ (PGD_2_) production in human mast cells in the presence of IL-33 (95). TSLP promoted MRGPRX2-triggered degranulation of human skin mast cells (96, 97).

A notable interspecies divergence between human and murine basophils pertains to their responsiveness to TSLP. In line with previous studies (57, 98), we confirmed that human basophils did not exhibit cytokine release (i.e., IL-4 and IL-13) upon exposure to TSLP (23). Moreover, TSLP stimulation also failed to induce CXCL8 secretion in human basophils. In contrast, murine basophils responded to TSLP with upregulation of mRNA expression and subsequent release of IL-4, IL-13, CXCL1, and CXCL2 (23). These results reinforce the role of TSLP in promoting the differentiation and activation of basophils in various mouse models (24, 36, 99, 100). TSLP induced chemotaxis and the formation of eosinophil DNA extracellular traps from human eosinophils (101, 102). This observation is relevant because there is emerging evidence that eosinophils and their DNA extracellular traps play a role in cancer initiation and growth (103, 104).

**Figure 3:**
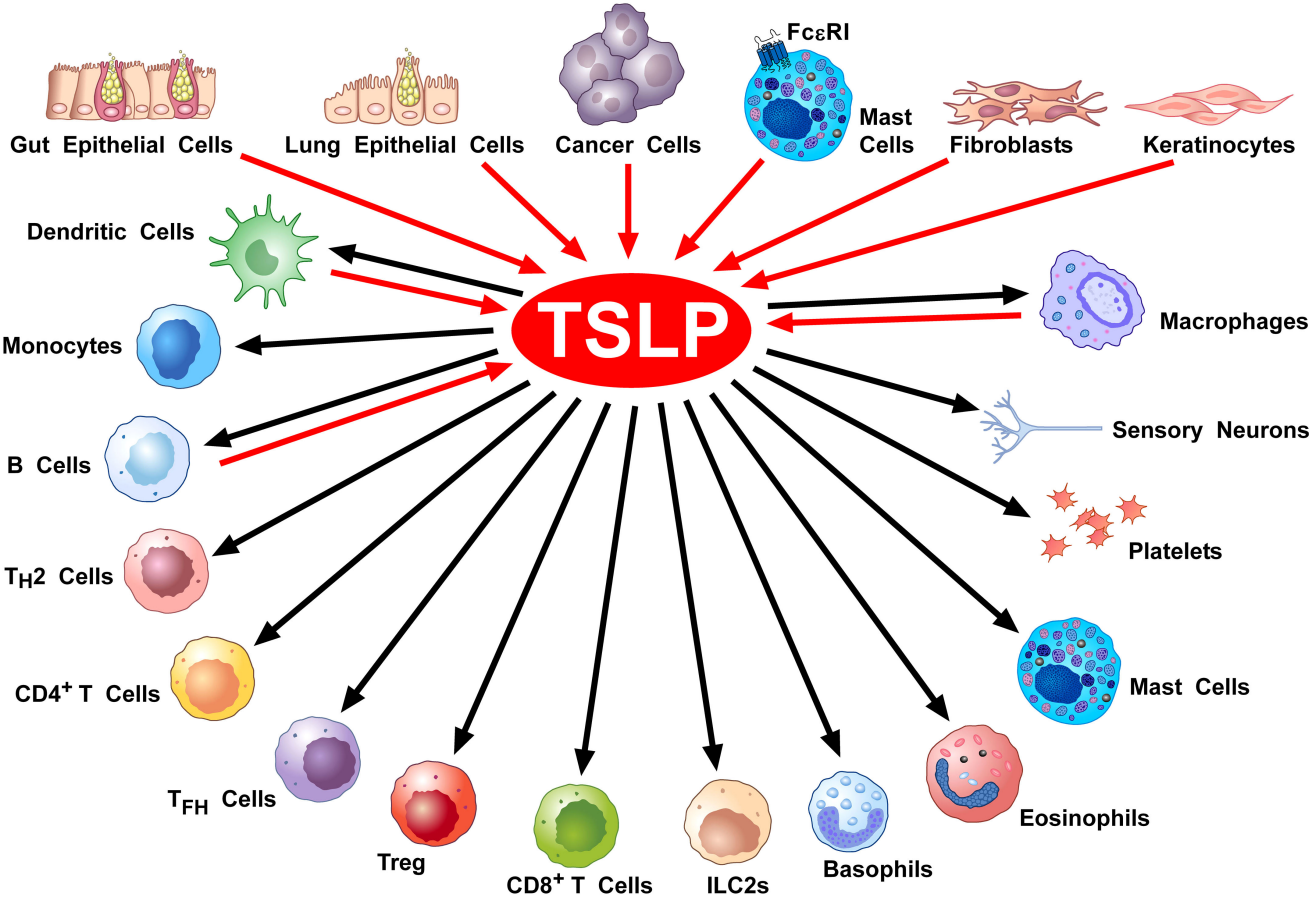
Cellular Sources and Targets of TSLP. A diverse array of triggers can activate lung (28, 29, 91, 105–107) and gut epithelial cells (66, 108–111), keratinocytes (30, 68, 70, 71, 112, 113) and cancer cells (114–118) to release TSLP. This alarmin can also be produced by mast cells (33, 92, 119, 120), DCs (121, 122), lung macrophages (48, 56, 74), and monocytes (56). Tryptase, released by mast cells can activate the protease-activated receptor 2 (PAR2) on fibroblasts (123, 124) and keratinocytes (123) to release TSLP. TSLP activates DCs (11, 63, 75, 76), CD4^+^ T and Th2 cells (18, 51, 77), ILC2 (63, 80, 81), NKT cells (125), CD8^+^ T cells (78, 126) and B cells (4, 82), Treg cells (86–88), murine (24) but not human basophils (23, 57), mast cells (91, 93–95), eosinophils (101, 102), macrophages (48, 55, 74), monocytes (48, 89), platelets (127, 128), and sensory neurons (123).

**Figure 3** shows the constellation of immune and structural cells that can be activated by TSLP.

## Protumorigenic role of TSLP in hematologic cancers

As previously emphasized, TSLP exerts several pleiotropic effects on cells of innate and adaptive immune system (40) that are directly and/or indirectly involved in the initiation and progression of tumors, angiogenesis and lymphangiogenesis (129–131). Hence, it is not surprising that TSLP would have a significant direct or indirect role in the regulation of experimental and human cancers (39–41).

**Figure 4:**
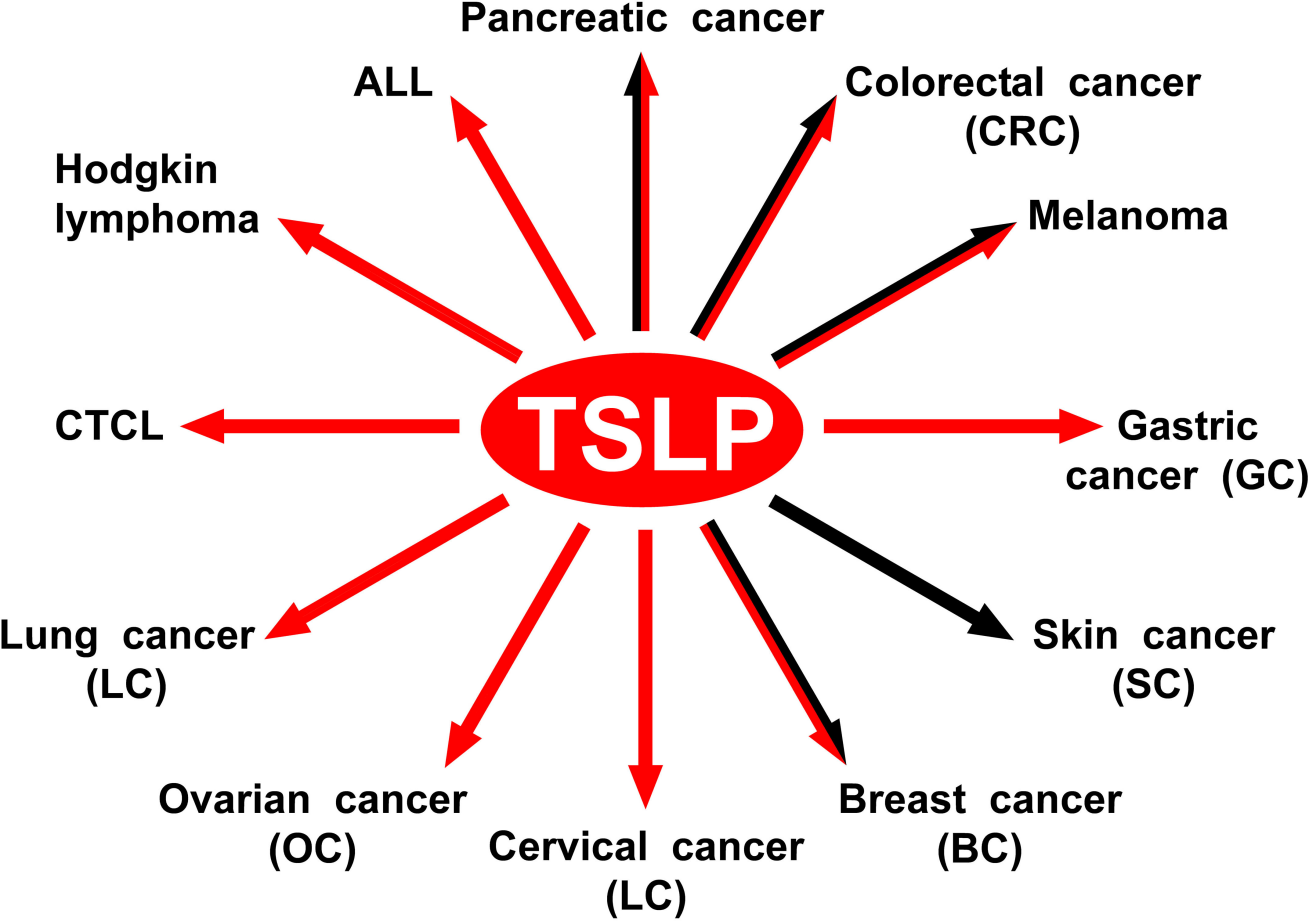
The protumorigenic role of TSLP in different hematologic and solid cancers. The red arrows indicate the human tumors in which TSLP plays a protumorigenic role. The black arrows indicate the experimental tumors in which TSLP appears to play a protumorigenic role.

**Figure 4** schematically illustrates the protumorigenic role of TSLP in different hematologic and solid cancers.

The cytokine receptor-like factor 2 (*CRLF2*) locus encodes for human TSLPR (7). Russell et al. first identified genetic rearrangements and mutations in the *TSLPR* gene in a percentage of pediatric patients with acute lymphoblastic leukemia (ALL) (132). Subsequent studies confirmed and extended the previous observation demonstrating rearrangement of *CRLF2* in approximately 15% of both pediatric and adult B-cell ALL (133, 134). A more recent study found *CRLF2* rearrangement in approximately 50% of pediatric B-cell ALL (135). In this study, TSLPR was absent in normal precursor B cells, but variably expressed in B-cell ALL by flow cytometry (135, 136). Rearrangements including deletions and translocations of TSLPR can be associated in the majority of B-ALL with activating mutations in the gene encoding the tyrosine kinase JAK2, which signals downstream of the TSLP receptor complex (133–135, 137). TSLP enhanced proliferation of long-term cultures of B-ALL cells (136). *CRLF2* overexpression was associated with a poor prognosis among children and adults with B-cell ALL (133, 134, 137–140). A recent study reported *CRLF2* rearrangement in 30% of Russian children with B-cell ALL and 72% of *CRLF* ^+^ were *TSLPR^+^* by flow cytometry (141). Approximately 80% *CRLF2* rearranged patients had translocation involving *P2RY8*, a known indicator of relapse in ALL. A study in a large cohort of 630 pediatric Chinese patients with B-ALL reported a low percentage of *P2RYB-CRLF2* (3.33%) and *CRLF2* (5.71%) overexpression. *P2RYB-CRLF2* identified only a subset of pediatric patients with poor prognosis (142).

TSLP concentrations are increased in plasma and overexpressed in lymph nodes of patients with Hodgkin lymphoma (143). TSLP mRNA is overexpressed in lesional skin and cutaneous T-cell lymphoma (CTCL) (144). TSLP induced the production of Th2 cytokines (e.g., IL-4 and IL-13) from CTCL cell lines and proliferation of CTCL cells through the activation of STAT5.

Studies supporting the protumorigenic role of TSLP in hematologic cancers are outlined in **Table 1**.

**Table 1:** Protumorigenic role of TSLP in hematologic cancers.

Cancer Type	Mechanisms	References
Pediatric Acute Lymphoblastic Leukemia (ALL)	Genetic rearrangements and overexpression of TSLPR gene (*CRLF2*).	(132)
Pediatric and Adult B-cell ALL	Genetic rearrangements and overexpression of *CRLF2* in approximately 14% of patients.	(133, 134)
Pediatric B-cell ALL	Genetic rearrangements in approximately 50% of patients.	(135)
B-cell ALL	TSLP enhanced proliferation of B-ALL cells.	(136)
Pediatric and Adult B-cell ALL	*CRLF2* overexpression was associated with poor prognosis.	(134, 137–139)
Pediatric B-cell ALL	*P2RY8-CRLF2* rearrangement was associated with poor prognosis.	(133, 138)
Pediatric B-cell ALL	*CRLF2* rearrangements in approximately 30-40% of patients. 80% of rearranged patients had translocation involving *P2RY8*.	(140, 141)
Pediatric B-cell ALL	*CRLF2* rearrangements in approximately 6% of patients. *P2RY8-CRLF2* overexpression in approximately 3% of patients.	(142)
Hodgkin Lymphoma	TSLP mRNA overexpression in lymph nodes.	(143)
Cutaneous T-cell lymphoma	TSLP mRNA overexpression in lesional skin.	(144)

## Protumorigenic role of TSLP in solid cancers

### Pancreatic cancer

Pancreatic cancer is a very aggressive disease characterized by a predominant Th2 (GATA3^+^) lymphoid infiltrate (145). Protti and collaborators first demonstrated that human pancreatic cancer [pancreatic ductal adenocarcinoma (PDAC)]-derived TNF-α and IL-1 induced the release of TSLP from cancer-associated fibroblasts (CAFs) (146). This observation was extended showing that TSLP released from CAFs activated TSLPR^+^ DCs to drive Th2 differentiation mediated by IL-4 released from basophils (147). The translational relevance of these findings was provided showing that *IL4* expressing basophils increased in tumor-draining lymph nodes (TDLN) of PDAC patients (148). Basophils in TDLN correlated with Th2 phenotype in tumors and were a negative prognostic marker of patient survival. Studies in a mouse model of pancreatic cancer confirmed a role for basophils during pancreatic cancer progression (147). Collectively, these results demonstrate that TSLP released from CAFs activates DCs, which induce T cells to secrete IL-3. Monocytes resident in TDLN secrete CCL7, which recruits basophils that are activated by IL-3 to release IL-4. This cytokine favors GATA3 expression in Th2 cells. A recent study identified IL-1α and IL-1β released by pancreatic cancer cells and tumor-associated macrophages as relevant stimuli for TSLP release from CAFs (149). The protumorigenic role of TSLP in PDAC was extended by showing that TSLP levels are detected *in situ* in tumor cells and systematically in advanced cancer patients (150). Moreover, elevated plasma TSLP concentrations were correlated with reduced overall patient survival (150). Although basophils account for 1% or less of the circulating leukocytes both in humans and mice, they have the propensity to infiltrate into the sites of inflammation (151). Basophils share some morphological and functional characteristics with mast cells, but these cells are distinct in many aspects (152). TSLP influences the development (24, 100) and activation of mouse basophils (23). Different models have uncovered unique roles for basophils in Th2 inflammatory responses (152–154) and parasitic infections (155–158). Moreover, there is growing evidence supporting the significant roles of basophils in cancer (159–163).

TSLP can induce mouse basophil maturation in an IL-3-independent manner and TSLP-elicited basophils *in vivo* were phenotypically distinct from IL-3-elicited basophils (24). TSLP caused the production of these cytokines/chemokines (IL-4, IL-13, CXCL1, and CXCL2) from mouse basophils (23), but did not induce cytokine release from human basophils (23). Basophils are present in the tumor microenvironment (TME) of various human (148, 161, 164, 165) and mouse experimental cancers (99, 148, 165, 166). Their involvement is increasingly recognized as influential in the onset and progression of both solid tumors and hematologic cancers (159, 162, 163, 167).

These cells play protumorigenic roles through different mechanisms. TSLP-activated mouse and human basophils are a major source of IL-4 and IL-13 (23, 57), which favor the polarization towards Th2 and M2 phenotypes (168). Moreover, basophils can release vascular endothelial growth factor-A (VEGF-A) (169) and cysteinyl leukotriene C_4_ (LTC_4_) (170, 171), which are implicated in the mechanisms of angiogenesis, tumorigenesis, and metastasis (172, 173).

### Breast cancer

Breast cancer is the most common malignancy in women and the second leading cause of cancer-related mortality in females (174). Mouse and human breast cancer cells express TSLP, which promotes Th2 differentiation of CD4^+^ T cells (175). Human breast cancer is heavily infiltrated by Th2 cells driven by OX40L-expressing DCs in response to cancer-derived TSLP (115). In a mouse model of breast cancer, TSLP activated resident macrophages to release VEGF-A, the most potent proangiogenic factor (118). Macrophages are a major anatomical and functional component of the TME, where they either promote or inhibit tumorigenesis and metastasis depending on their functional state (176, 177).

For decades, macrophages were simplistically classified into two groups, referred to as “classically activated M1” or “alternatively activated M2” endotypes (168). M2-like phenotype is mostly the phenotype of tumor-associated macrophages (TAMs) (168). Different subpopulations of TAMs promote angiogenesis, tumor invasion, suppress cytotoxic T-cell responses and promote the formation of metastasis (178). Single-cell analyses have identified several subsets of TAMs in human cancers (165). T2 cytokines (i.e., IL-4 and IL-13) drive the differentiation of macrophages into alternatively activated macrophages (131, 179). TSLP changes the phenotype of macrophages toward an M2-like phenotype during TSLP-induced airway inflammation (55). This differentiation of macrophages was IL-13-, but not IL-4-dependent. These results demonstrate that TSLP/TSLPR plays a significant role in the amplification of alternatively activated macrophage polarization (55).

Kuan and Ziegler demonstrated that TSLPR is expressed by human breast cancer cells and mouse TAM expressed TSLP (117). Interestingly, non-tumor breast tissue did not express TSLPR. Moreover, *Tslp* mRNA was increased in TAM, monocytes, and neutrophils from both breast cancer patients and mice. They also demonstrated that TSLP from non-tumor derived sources (i.e., IL-1α-activated neutrophils) is critical for breast tumor metastasis in lungs (117). The authors concluded that a breast-myeloid cell axis, mediated *via* TSLP and IL-1α, promotes the progression of breast cancer and metastasis formation (117).

Activation of primary breast cancer tissues, as well as surrounding tissue, released several proinflammatory cytokines (i.e., IL-1α, IL-1β, IL-18, and IL-33) (116). The secretion of cytokines was higher in breast cancer tissues than in non-malignant ones. cCD11c^+^ myeloid cells, including monocytes and DCs, were the main source of IL-1β in human breast cancer. IL-1β selectively induced TSLP secretion from breast cancer cells. These findings suggest that Th2 inflammation in breast cancer is dependent on IL-1β *via* TSLP induction. Importantly, neutralization of IL-1β prevented breast cancer progression in a humanized mouse model (116).

In a mouse model, TSLP released from breast cancer downregulates the receptors, CXCR4 and α4β1 integrin, which physiologically keep B-cell precursors in bone marrow (84). Using mouse and human bone marrow aspirates incubated with metastatic 4T1 breast cancer cells, the authors demonstrated that this was the result of TSLP release from cancer cells. The loss of CXCR4 signaling or α4β1 integrin binding to VCAM-expressing stromal cells, caused the exit of B-cell precursors from the bone marrow. It was suggested that these cells can differentiate into Bregs or suppressive B cells in TME, favoring lung metastasis (84). Finally, TSLP is overexpressed by immunohistochemistry in breast cancer compared to normal breast tissue and is associated with an increased risk in breast cancer in Saudi women (180).

### Melanoma

Malignant melanoma continues to be a major health concern despite the developments of immunotherapy and targeted therapy (181, 182). Yao and collaborators used genetically engineered models of melanoma and tumor cell grafting combined with TSLP knockout or overexpression, to identify a crosstalk between keratinocytes, immune cells, and melanoma cells in TME (183). Melanoma cell-derived factors in *Braf/Pten* mice activated keratinocytes to release TSLP, which engaged TSLPR on DCs. These cells promoted the activation of GATA3^+^ Foxp3^-^ Th2 cells to release IL-4 and IL-13. At the same time, TSLP-activated DCs promoted GATA3^+^ Foxp3^-^ Treg cells showing suppressive activity on CD8^+^ T cell proliferation and IFN-γ production. Interestingly, a similar population of GATA3^+^ Tregs was also found in human melanoma. A similar subset of GATA3^+^ Tregs was also found in skin biopsies from patients with primary human melanoma. This study highlights the role of TSLP in programming a protumoral immune microenvironment in melanoma (183). Collectively, these results highlight a novel circuit involving keratinocytes-derived TSLP, which activates DCs and CD4^+^ cells to release IL-4 and IL-13, promoting the growth and metastasis of melanoma (183).

Eosinophils are present in the TME of several human solid (184–188) and hematologic tumors (189), and experimental cancers (190). Eosinophils release a plethora of mediators that individually have positive or negative effects on various immune cells (191). Studies addressing the potential functions of eosinophils in experimental and human tumors have provided conflicting results (192–194). In experimental studies, a protective role of eosinophils was found in melanoma (23, 195–199), Hodgkin’s lymphoma (200), hepatocellular carcinoma (201), and prostate cancer (202). IL-33 administration in mice-bearing melanoma resulted in tumor growth delay and prevented pulmonary metastasis (196, 199). On the other side, human eosinophils produce several proangiogenic factors such as VEGF-A (203), fibroblast growth factor (FGF-2) (195, 204), and CXCL8/IL-8 (205). Eosinophils release chemokines (CCL5, CCL9, CXCL10) important for the attraction of CD8^+^ T cells in TME (195).

Association studies have revealed that a higher presence of basophils (i.e., CD123^+^, CCR3^+,^ FcεRI^+^) within tumors is correlated with improved overall survival (161). In a mouse melanoma model, basophils released CCL3 and CCL4, which played a crucial role in attracting CD8^+^ T cells to the tumor site, thereby promoting tumor rejection (161, 206, 207). Although the mechanisms by which basophils contribute to tumor suppression are not fully understood, certain mediators (e.g., granzyme B and TNF-α) released by these cells have tumor-killing properties. Moreover, basophils secrete chemokines (e.g., CCL3 and CCL4) involved in attracting cytotoxic CD8^+^ T cells into the TME (163).

### Colorectal cancer

Colorectal cancer (CRC) is the third most common type of cancer and the second leading cause of malignancy-related mortality among the global population (208). Obata-Ninomiya and collaborators analyzed six independent databases and found that TSLP expression correlated with CRC and was a marker of poor prognosis (209). The expression of TSLP mRNA in colon cancer tissue was increased compared to normal colon from the same patients (209, 210). These findings were extended by showing increased expression of TSLP, TSLPR, and IL-7Rα by immunohistochemistry in colon cancer tissues compared to normal colon. The authors also found that TSLP rs10043985 polymorphism was strongly correlated with CRC in Saudi patients (210). The latter finding suggests that this mutation in the promoter region of *TSLP* might play a detrimental role in CRC.

In a mouse model of colitis associated with CRC, TSLP mRNA was overexpressed in colon cancer compared to non-tumor sites and control mice (209). The number of tumors in *Tslp^-/-^* mice was reduced compared to *Tslp^+/+^* mice, suggesting that TSLP plays a protumorigenic role in this model of CRC. The frequency of Treg expressing TSLPR (TSLPR^+^ Tregs) was increased in colon cancer and TSLPR^+^ Tregs exhibited stronger immunosuppressive activity compared to TSLPR^-^ Tregs *in vitro* and *in vivo*. TSLPR^+^ Tregs subset coexpressed ST2, CTLA-4, PD-1 that are associated with CRC in humans and mice (211–213). Collectively, these results indicated that TSLPR^+^ ST2^+^ Treg subset was involved in CRC development and progression (209). Although ST2 detection on Tregs had no effect on tumor number and size, double deficiency of TSLPR and ST2 on Tregs reduced tumor progression. These results suggested that TSLPR signaling rather than ST2 signaling by TSLPR^+^ ST2^+^ Tregs is important in tumor growth. The latter finding suggested that TSLPR blockade signaling could be effective for the treatment of CRC. In fact, the administration of an anti-TSLP monoclonal antibody reduced the size and number of CRC (209). This treatment was associated with decreased TSLPR^+^ ST2^+^ Tregs in colon and lymph nodes and increased Th1 cells in colon. Collectively, these findings demonstrate for the first time that an anti-TSLP antibody is effective in a mouse model of colitis-associated CRC.

These results have translational relevance in colorectal tumors in humans. The frequency of intratumor TSLPR^+^ ST2^+^ Foxp3^+^ CD25^hi^ Tregs was increased in patients with CRC, compared to adjacent normal colon from the same donor. The frequency of this Tregs subset was also increased in peripheral blood from these patients (209). These results are consistent with those observed in the murine model supporting the notion that TSLPR^+^ ST2^+^ Tregs promote a protumorigenic microenvironment during CRC initiation and progression.

### Lung cancer

Lung cancer is the leading cause of cancer mortality in men and the second in women, behind breast cancer (214, 215). Non-small cell lung cancer (NSLC) comprises 85% of lung cancers and 40% of those are adenocarcinomas (216). The human lung is particularly rich in a variety of cells of innate and adaptive immune system (217, 218), and tumor-infiltrating myeloid cells are key regulators of lung cancer initiation and progression (217, 219).

TSLP expression, examined by immunohistochemistry, was increased in intratumoral lung cancer compared to non-cancer tissue and benign lesions (220). The number of Foxp3^+^ Tregs in lung cancer tissue was increased compared to non-cancer tissue, particularly in the group of TSLP^+^ cancers. TSLP induced the differentiation of CD4^+^ CD25^-^ T cells into Tregs (220). Recently, we have found that TSLP, TSLPR, and IL-7Rα expression, examined by immunohistochemistry, was higher in the intratumoral lung cancer compared to the peritumoral area (56). Total TSLP protein was also increased in intratumoral compared to peritumoral lung tissue. We also examined the expression of the two TSLP isoforms (lfTSLP and sfTSLP), TSLPR, and IL-7Rα mRNAs in peritumoral and intratumoral lung cancer. The proinflammatory lfTSLP mRNA was higher in peritumoral tissue, whereas the sfTSLP mRNA was overexpressed in intratumoral compared to peritumoral lung cancer. The TSLPR mRNA was equally expressed in both compartments. The IL-7Rα mRNA was highly expressed in intratumoral lung tissue (56). These results provide the first evidence that the protein and molecular expression of the different components of the TSLP/TSLPR network differ at the intra- and peritumoral levels in cancer. Furthermore, these results provide the first demonstration that the molecular expression of the two isoforms of TSLP is differentially expressed at peri- and intratumoral levels in human lung cancer. These results suggest that the expression and the pathogenic role(s) of the two isoforms of TSLP should be carefully investigated in the initiation and progression of other human cancers.

In the same study, it was demonstrated that macrophages purified from macroscopically normal lung parenchyma of patients with lung cancer constitutively express TSLP, TSLPR, and IL-7Rα (56). Activation of human lung macrophages (HLMs) with IL-4, alone and in combination with IL-13, induced the overexpression of lfTSLP mRNA and TSLP release (56). Moreover, lipopolysaccharide (LPS), a promoter of metastatic cells (221), was a potent stimulus for the release of TSLP from HLMs. Finally, LPS synergistically potentiated TSLP release induced by IL-4 from HLMs (56). More recently, it was demonstrated that TSLP, but not sfTSLP, can activate HLMs to release VEGF-A, the most potent angiogenic factor. Interestingly, sfTSLP did not induce nor interfere with the activating property of lfTSLP on HLMs (48). These results unveil an intriguing interplay between TSLP and HLMs that might be relevant in lung cancer. Th2-like cytokine in TME and LPS can induce TSLP release from HLMs. TSLP, but not sfTSLP, can feedback on TSLPR on HLMs to induce the release of angiogenic factors that can contribute to lung cancer growth. In conclusion, TSLP released by lung macrophages can play a role in the autocrine circuit that could favor lung cancer progression.

Human basophils co-cultured with the human lung adenocarcinoma cell line A549, release copious amounts of IL-4 and IL-13 (98). In human and mouse NSCLC, IL-4 derived from bone marrow basophils and eosinophils promoted the development of immunosuppressive tumor-promoting myeloid cells (162). Depletion of basophils and the administration of dupilumab, IL-4Rα blocking antibody (222), reduced tumor growth (162). Collectively, these results further suggest that basophils may contribute to tumor progression through the release of copious amounts of Th2-like cytokines (163, 223).

### Gastric cancer

Gastric cancer is the fifth most prevalent malignancy and the fourth leading cause of cancer death worldwide (224). TSLP mRNA was overexpressed in the majority of gastric cancer patients compared to distant tumor-free samples (225). A significant association was reported between TSLP overexpression and lymph node metastasis. In another study, the expression of TSLP examined by immunohistochemistry was higher in cancer tissue compared to non-tumor sites (226). Higher tissue expression of TSLP and higher circulating levels of this cytokine were associated with a poor prognosis of gastric cancer (226).

### Cervical cancer

Cervical cancer is one of the most common gynecological malignancies with high rates of morbidity and mortality (227). TSLP examined by immunohistochemistry was overexpressed in human cervical cancer compared to cervicitis (114). Cervical carcinoma HeLa and CaSki cells released TSLP *in vitro*. TSLP induced proliferation of human umbilical vein endothelial cells (HUVEC) expressing TSLPR and cervical carcinoma cell-derived TSLP promoted HUVEC proliferation. The authors concluded that TSLP released from human cervical cancer can promote tumor angiogenesis through the activation of TSLPR on endothelial cells (114). This group extended the previous findings showing that TSLP released from cervical cancer cells can activate eosinophils to produce proinflammatory cytokines (187). A more recent study reported that TSLP stimulates the proliferation and invasion of HeLa and SiHa cells by downregulating the expression of miR-132 (228).

### Skin cancer

Human (68, 112, 229) and mouse keratinocytes (113) are a major source of TSLP. In a mouse model, repeated topical exposure to environmental carcinogens induced skin inflammation and enhanced the circulating and local levels of polyclonal IgE (99). IgE increase was accompanied by skin infiltration of basophils releasing Th2 cytokines (IL-4, IL-6, and IL-13). Basophil-derived conditioned media promoted proliferation of epithelial cells and the expression of inflammatory cytokines (i.e., IL-1α, IL-18, and IL-31). Basophil recruitment to the inflamed skin was dependent on TSLP/IL-3-mediated upregulation of CXCR4 in basophils (99). TSLP, abundantly expressed in inflamed skin, induced the transport of CXCR4 to the basophil surface. These results suggest that TSLP and IL-3 produced at site of skin inflammation drive the expression of CXCR4 on basophils, allowing recruitment to the skin in response to increased levels of CXCL12. In this model of inflammation-driven epithelial carcinogenesis, TSLP plays a key role in the promotion of epithelial hyperplasia and tumor growth (99).

### Ovarian cancer

TSLP mRNA was overexpressed in human epithelial ovarian carcinoma (EOC) compared to adjacent normal tissues (230). TSLP protein overexpression was found in approximately 60% of 144 patients with EOC and 16% of benign cases. Patients with TSLP overexpression were associated with worse survival and lower overall survival (OS) (230). It has been reported that sfTSLP mRNA was selectively expressed by human ovarian cancers (231). Overexpression of sfTSLP in TSLP ovarian and endometrial cancer cells promoted tumor growth *in vitro*. The authors concluded that sfTSLP was predominantly expressed in human ovarian cancers and promoted tumor growth *in vitro*. These intriguing results emphasize the need for further studies to investigate the expression and role(s) of the two TSLP isoforms in human cancers.

Studies supporting the protumorigenic role of TSLP in human and experimental solid cancers are outlined in **Table 2**.

**Table 2:** Protumorigenic role of TSLP in solid cancers.

Cancer Type	Model	Mechanisms	References
Pancreatic cancer	Human/Mouse	TNF-α and IL-1β induced TSLP release from cancer-associated fibroblasts (CAFs). TSLP activated TSLPR^+^ DCs.	(146, 147)
Pancreatic cancer	Mouse	IL-1α and IL-1β from pancreatic cancer cells released TSLP from CAFs.	(147, 149)
Pancreatic cancer	Human	TSLP was detected *in situ* in cancer cells and plasma levels were correlated with poor prognosis.	(150)
Breast cancer	Human/Mouse	Breast cancer cells and tumor-associated macrophages expressed TSLP. Breast tumor cell-derived IL-1α induced TSLP expression in several immune cells. TSLP was critical for experimental breast tumor metastasis.	(117)
Breast cancer	Human/Mouse	Breast cancer cells expressed TSLP.	(175)
Breast cancer	Human	Breast cancer cells released TSLP.	(115)
Breast cancer	Mouse	TSLP induced VEGF-A release from cancer resident macrophages.	(118)
Breast cancer	Human	IL-1β induced TSLP release from breast cancer cells.	(116)
Breast cancer	Mouse	TSLP released from breast cancer cells promoted lung metastasis.	(84)
Melanoma	Mouse/Human	Keratinocyte-derived TSLP promoted growth and metastasis of melanoma programming a suppressive tumor microenvironment.	(183)
Colorectal cancer	Human/Mouse	TSLP expression correlated with poor prognosis in colorectal cancer (CRC).	(209)
Colorectal cancer	Mouse	TSLP mRNA was overexpressed in cancer tissue. Tregs expressing TSLPR were increased in CRCs and were associated with progression of CRCs in human. A monoclonal antibody anti-TSLP reduced the size and number of CRC in mice.	(209)
Colorectal cancer	Human	TSLP mRNA was overexpressed in colon cancer.	(209, 210)
Colorectal cancer	Human	TSLP rs10043985 polymorphism was correlated with CRC.	(180)
Lung cancer	Human	TSLP was overexpressed in intratumoral lung cancer and correlated with Foxp3^+^ Tregs.	(220)
Lung cancer	Human	TSLP, TSLPR, and IL-7Rα were overexpressed in intratumoral lung cancer. lfTSLP and sfTSLP were differently expressed in peritumoral and intratumoral lung cancer tissues.	(56)
Lung cancer	Human	lfTSLP but not sfTSLP activated human lung macrophages to release VEGF-A.	(48, 56)
Lung cancer	Human	Basophils co-cultured with human lung adenocarcinoma A549 released IL-4 and IL-13.	(98)
Gastric cancer	Human	TSLP mRNA was overexpressed in intratumoral gastric cancer.	(225, 226)
Gastric cancer	Human	Higher tissue expression and circulating levels of TSLP were associated with poor prognosis.	(226)
Cervical cancer	Human	TSLP was overexpressed in cervical cancer. Cervical carcinoma cell lines released TSLP, which promoted endothelial cell proliferation.	(113)
Skin cancer	Mouse	Skin carcinogens induced basophil recruitment to the skin mediated by TSLP.	(165)
Ovarian cancer	Human	TSLP was overexpressed in ovarian cancer and associated with poor prognosis.	(227, 228)

**Figure 5:**
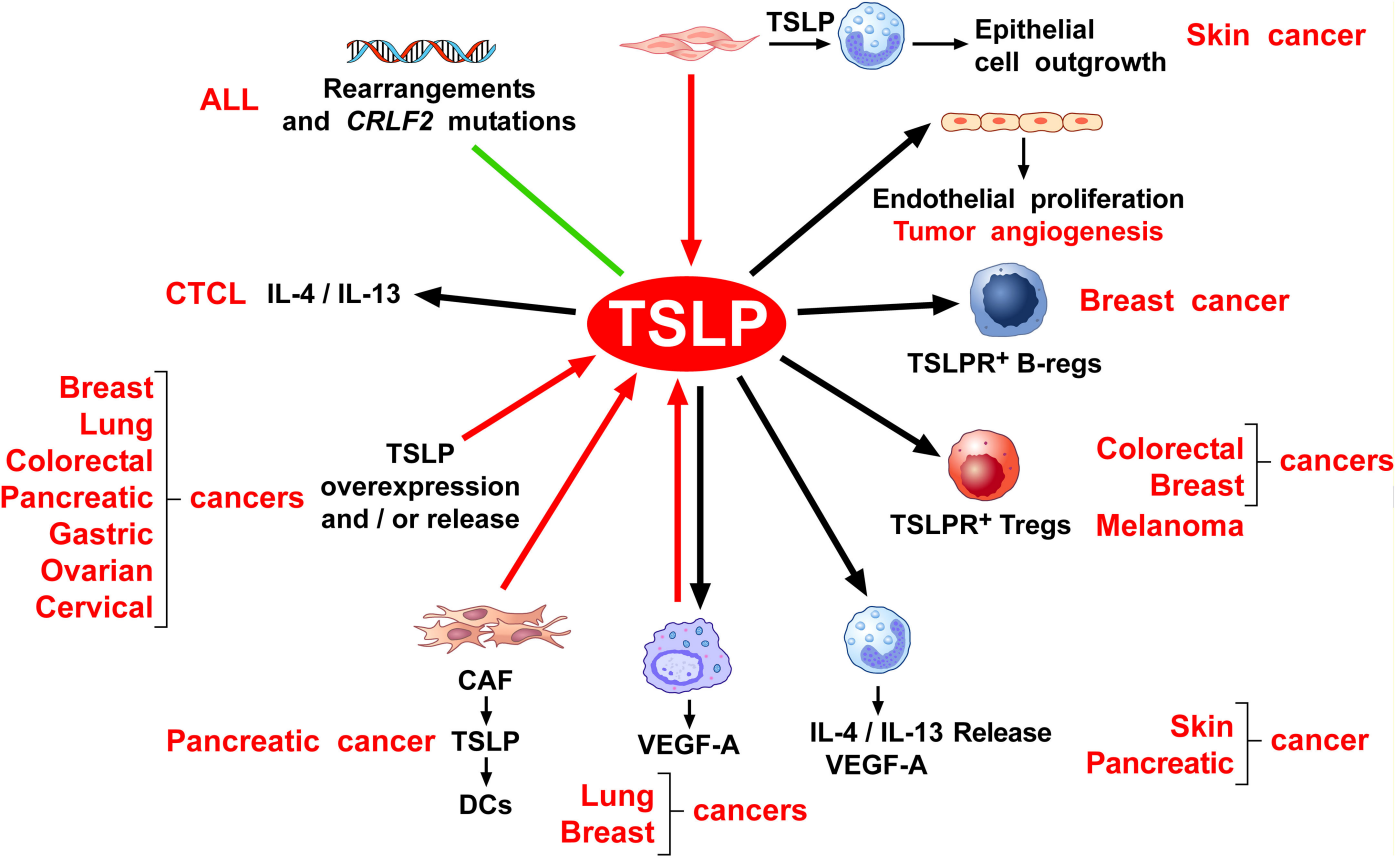
Possible mechanisms by which TSLP may play a protumorigenic role. Several lines of evidence suggest that thymic stromal lymphopoietin (TSLP) contributes to tumor development and progression through various mechanisms. Rearrangement and mutation of the cytokine receptor-like factor 2 (*CRLF2*) locus which encodes for human TSLPR are found in a variable percentage of children and adult patients with acute lymphoblastic leukemia (ALL) (132–142). TSLP induces the production of Th2 cytokines (e.g., IL-4, IL-13) from cutaneous T-cell lymphoma (CTCL) (144), thereby contributing to a protumorigenic immune milieu. Beyond hematologic malignancies, TSLP has also been implicated in a variety of solid tumors. Several human (56, 114, 117, 150, 175, 187, 209, 210, 225, 230) and mouse cancers (209) overexpress and/or release TSLP. Within the tumor microenvironment (TME), TSLP released from cancer-associated fibroblasts (CAFs) from pancreatic cancer activates TSLPR^+^ DCs to drive Th2 and macrophage M2 phenotypes (146, 148), contributing to a protumorigenic immune microenvironment. Similarly, tumor-associated macrophages (TAMs) from lung cancer patients express and release TSLP (48, 56, 74). Once activated by TSLP, TAMs release vascular endothelial growth factor-A (VEGF-A), a key mediator of angiogenesis (48, 56). Consistently, in a mouse model of breast cancer, TSLP can activate macrophages to release VEGF-A (118). TSLP can also exert direct pro-angiogenic effects. In human cervical cancer, TSLP can promote tumor angiogenesis through the activation of TSLPR^+^ endothelial cells (114). Additionally, TSLPR^+^ Tregs exhibit strong immunosuppressive activity in both human and experimental models of colorectal (209) and breast cancer (175) and melanoma (183), helping tumors evade immune surveillance. In a mouse model, TSLP released from breast cancer cells promotes the differentiation of B-cell precursors into Bregs or immunosuppressive B cells in tumor microenvironment (TME) (84). Furthermore, both mouse and human basophils activated by TSLP are a major source of T2 cytokines (IL-4, IL-13) (23, 57), which promote Th2 and M2-skewed immune responses (168). Finally, keratinocyte-derived TSLP promotes growth and metastasis of human and experimental melanoma by activating TSLPR^+^ DCs which induce Tregs and immunosuppression in TME (183). In a mouse model of chronic skin inflammation, basophils are recruited to inflamed skin *via* TSLP (99), promoting epithelial cell outgrowth harboring oncogenic mutations.

**Figure 5** schematically illustrates the possible mechanisms by which TSLP plays a protumorigenic role in different human and experimental cancers.

## Antitumorigenic role of TSLP in solid cancers

### Breast cancer

In a mouse model, TSLP overexpression in the skin leads to inflammation, which was associated with inhibition of early stages of breast carcinogenesis (232). TSLP-induced breast cancer suppression was associated with CD4^+^ T cell accumulation around breast cancer (232). The same group also examined the possible role of TSLP induction during breast cancer development using the PyMt cell line model in *Tslp* ^tg^ mice (233). In an orthotopic breast tumor model, primary breast cancer cells from PyMt^tg^ mice or PyMt cell line were implanted into the mammary fat pad of *Tslp^tg^* and wild-type (WT) controls. *Tslp^tg^* mice receiving PyMt primary cells had delayed tumor growth and smaller tumors compared with WT mice. *Tslp^tg^* mice receiving PyM cell line also showed delayed tumor growth. Analysis of PyMt cell line-derived breast tumor revealed increasing CD4^+^ T cells in *Tslp^tg^* compared with WT mice. TSLP-activated CD4^+^ T cells sorted from the tumors inhibited the growth of PyMt cells *in vitro*. TNF-α and IFN-γ present in supernatants of TSLP-activated CD4^+^ T cells were required for PyMt tumor suppression. The authors concluded that TNF-α and IFN-γ produced by TSLP-stimulated CD4^+^ T cells play a major role in providing antitumor immunity against experimental breast cancer (233).

### Lung cancer

To evaluate the role of TSLP on early lung carcinogenesis, a mouse model of spontaneous lung adenocarcinoma, Kras^+/GI2D^ (Kras^GI2D^) was crossed with K14-TSLP^tg^ (Tslp^tg^) mice. Tslp^tg^ Kras^GI2D^ mice developed a lower lung tumor burden compared to Kras^GI2D^ mice. Tslp^tg^ Kras^GI2D^ lung tumors were composed of lower-grade atypical alveolar hyperplasia and adenoma compared to adenocarcinoma in Kras^GI2D^ lung (234). CD4^+^ T cell depletion inhibited the proliferative impact of TSLP against lung carcinogenesis in TSLP overexpressing mice. The authors suggested that in this experimental model of lung carcinogenesis, TSLP inhibits the early stages of lung cancer development.

### Skin cancer

In a mouse model of Notch-deficient skin carcinogenesis, it has been proposed that TSLP-mediated inflammation protects against carcinogenesis (235). TSLP-mediated tumor protection was mediated by CD8^+^ and CD4^+^ T cells. The protective effect of TSLPR signalling was also confirmed in a model of Notch-independent skin cancer (235). Demeri et al. extended the previous findings showing that Notch-deficient mice develop severe skin inflammation caused by epidermal TSLP overexpression. Blocking TSLP signalling in Notch-deficient animals resulted in skin carcinogenesis. The authors concluded that upregulation of epidermal TSLP can generate anti-tumor CD4^+^ T cell response in a Th2 inflammatory microenvironment (236). Studies in humans appear necessary to clarify the possible role of TSLP/TSLPR network in skin carcinogenesis.

### Endometrial cancer

Endometrial cancer is one of the most common types of gynecologic cancers worldwide (237). A recent study reported that the expression of TSLP (measured by Western blot) was reduced in several human endometrial cancer cell lines compared to normal human endometrial cells (238). Micrograms of TSLP partially inhibited the proliferation of two endometrial cancer cell lines. High concentrations of TSLP alone had no effect on the *in vitro* proliferation of an endometrial cancer cell line, but slightly enhanced the inhibitory effect of progesterone (238). The authors concluded that the loss of TSLP in endometrial gland epithelial cells may contribute to endometrial cancer development. The concentrations of TSLP used in these experiments exceed by several logarithms the pathophysiological levels of this cytokine making the results of difficult interpretation.

### Colon cancer

Yue et al. observed a reduction in TSLP expression in human colon cancer, and there was an inverse relationship between TSLP levels and the clinical stage of the cancer (239). TSLP promoted apoptosis of colon cancer cells through the engagement of TSLPR. Using a xenograft mouse model, the authors reported that peritumoral administration of TSLP reduced tumor growth.

Studies supporting the antitumorigenic role of TSLP in experimental and human cancers are outlined in **Table 3**.

**Table 3:** Antitumorigenic role of TSLP.

Cancer Type	Model	Mechanism	References
Breast cancer	Mouse	TSLP induced CD4^+^ T cell accumulation around breast cancer.	(232, 233)
Lung cancer	Mouse	TSLP inhibited the early stages of lung cancer development.	(234)
Skin cancer	Mouse	TSLP-mediated inflammation protects against skin carcinogenesis.	(232, 235)
Endometrial cancer	Human	High concentrations of TSLP inhibited endometrial cancer cell proliferation *in vitro*. Reduced expression of TSLP in endometrial gland epithelial cells.	(238)
Colon cancer	Human	High concentrations of TSLP inhibited colon cancer growth *in vitro.*	(239)

## Conclusions and future perspectives

**Figure 6:**
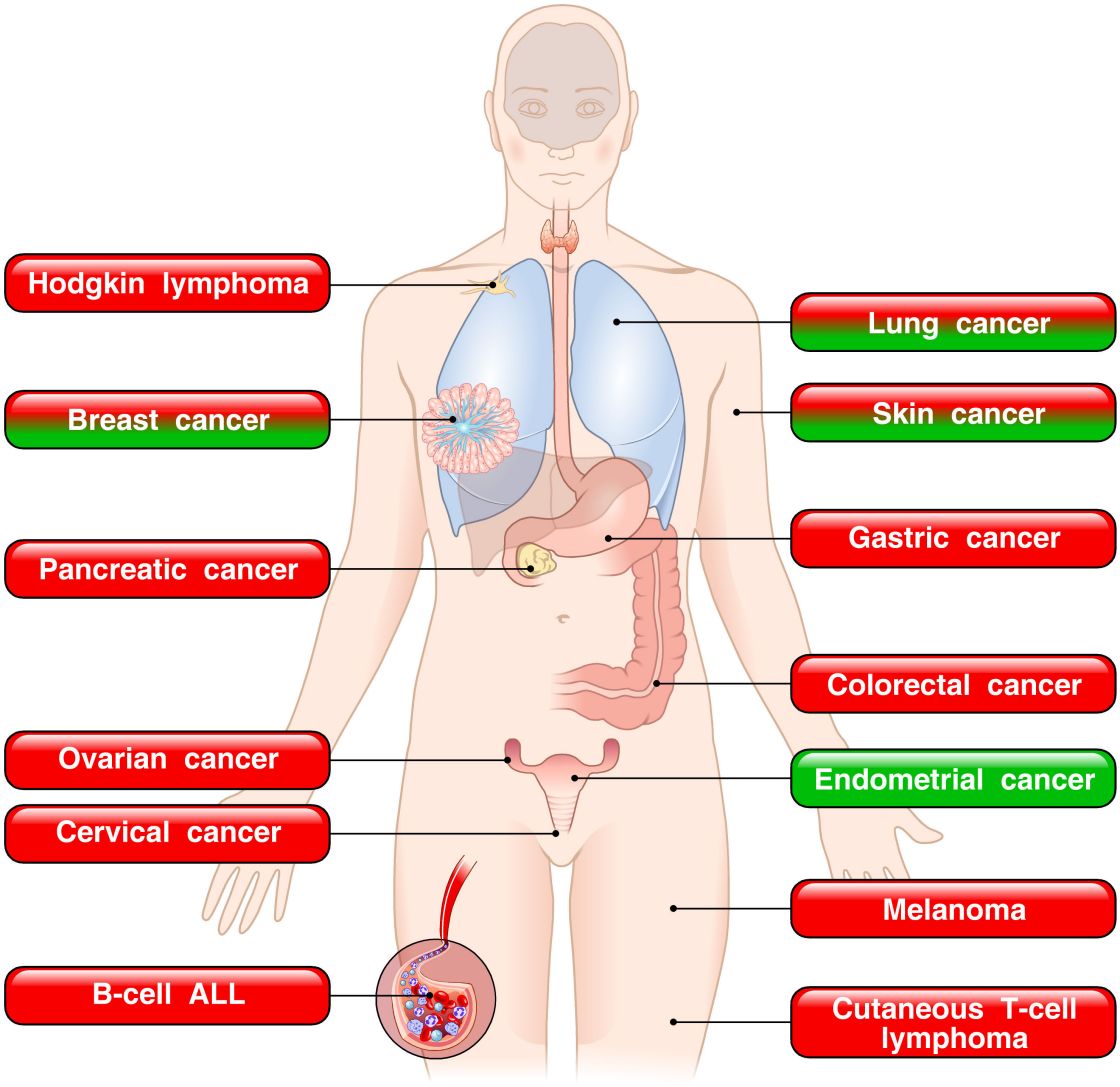
Dual role of TSLP in human tumors. The red boxes indicate the tumors in which TSLP is implicated in promoting tumor growth. The green boxes represent tumors in which TSLP appears to play a protective role. The mixed red/green boxes depict tumors in which TSLP plays both pro- and antitumorigenic roles in various experimental and human cancers.

Previous reviews started to highlight the controversial nature of the TSLP–TSLPR axis in both experimental models and human cancers (39–41). Since then, several clinical and experimental studies have extended the intriguing observation that in different neoplasias TSLP can play a protumorigenic role or protective effects depending on the tumor context. In human hematologic cancers, such as ALL, Hodgkin disease and CTCL, TSLP appears to promote tumor progression (**Figure 6**).

By contrast, in a variety of human solid cancers, TSLP can play a protumorigenic, an antitumorigenic role, or both (**Figure 6**). In the vast majority of cancers (pancreatic, ovarian, cervical, gastric, colorectal cancers and melanoma), TSLP has been found to promote cancer initiation and growth. By contrast, in a model of sex hormone-dependent endometrial cancer, TSLP seems to play a protective role (238). In this study, industrial concentrations of TSLP inhibited cancer cell proliferation (238). It is intriguing that in certain tumors (breast, lung and skin cancers), different studies reported opposing views of TSLP in tumorigenesis. A possible explanation of these apparently different results is that the majority of studies showing an antitumorigenic effect of TSLP were performed in different mouse experimental models (232–236). Notably, the protumorigenic effects of TSLP were demonstrated in several human and experimental models of different cancers. The development of appropriate and specific animal models appears necessary to better understanding of the underlying mechanisms of TSLP-driven tumorigenesis in cancers.

In human cancers, the role of TSLP isoforms, which occur only in this species, has not been thoroughly investigated. There is preliminary evidence that the two variants of TSLP (lfTSLP and sfTSLP mRNAs) are differentially expressed at peri- and intratumoral levels in human lung cancer (56). Moreover, there is some evidence that sfTSLP is selectively expressed in human ovarian cancer (231). These preliminary results demand that the roles of the two TSLP isoforms should be examined during the initiation and progression of other human cancers.

The results of several studies have suggested that TSLP can exert a protumorigenic role through different mechanisms. For instance, TSLP can favor Th2 and M2 polarization in several cancers, including pancreatic cancer (146–148), melanoma (183), skin cancer (99), breast cancer (115–117, 175), and CTCL (144). TSLP can also increase the frequency of Tregs (209) in experimental and human colorectal cancer (209) and melanoma (183). In breast cancer, the protumorigenic mechanism is dependent on IL-1β released by cancer cells that activate myeloid cells in TME. The latter cells release TSLP, which promotes tumor cell proliferation (116). Finally, it has been shown in a mouse model of breast cancer that TSLP can activate resident macrophages to release VEGF-A (118). We have extended the latter observation showing that TSLP, but not sfTSLP, can induce the release of VEGF-A and VEGF-C from macrophages isolated from patients with lung cancer (48, 56). There is also the possibility that TSLP released from cancer cells can directly activate endothelial cells expressing TSLPR (114).

From a translational perspective, a deeper understanding of the tumor context-dependent effects of TSLP isoforms may encourage the identification of reliable biomarkers to stratify patients who might benefit from therapeutic targeting of the TSLP–TSLPR axis. Indeed, the role of TSLP in cancer initiation and growth has significant implications, especially considering the recent approval of an anti-TSLP monoclonal antibody (tezepeleumab) for the treatment of asthma, a common inflammatory disease of the respiratory system (60). On one side, it has been demonstrated that the administration of an anti-TSLP antibody decreased colorectal cancer in a mouse model (209). On the other side, if TSLP plays an antitumorigenic role in certain tumors, the administration of biological therapies targeting TSLP/TSLP receptor network could lead to negative effects.

Finally, considering the proposed homeostatic and anti-inflammatory functions of sfTSLP (71), these characteristics warrant careful consideration in the development of targeted therapies for cancer initiation and progression. In conclusion, the above considerations emphasize the urgency of further investigating the role of TSLP and its isoforms in the onset and progression of human and experimental cancers. A deeper understanding of the immunological and molecular determinants driving the dual behavior of TSLP in the tumor microenvironment will be essential to support the development of precision immunomodulatory strategies in oncology.
